# High prevalence of canine heartworm, *Dirofilaria immitis*, in pet dogs in south Texas, USA, with evidence of *Aedes aegypti* mosquitoes contributing to transmission

**DOI:** 10.1186/s13071-022-05514-1

**Published:** 2022-11-03

**Authors:** Nicole A. Scavo, Italo B. Zecca, Caroline Sobotyk, Meriam N. Saleh, Sarah K. Lane, Mark F. Olson, Sarah A. Hamer, Guilherme G. Verocai, Gabriel L. Hamer

**Affiliations:** 1 Ecology and Evolutionary Biology, Texas A&M University, College Station, TX USA; 2 Department of Veterinary Integrative Biosciences, School of Veterinary Medicine and Biomedical Sciences, Texas A&M University, College Station, TX USA; 3 Department of Veterinary Pathobiology, School of Veterinary Medicine and Biomedical Sciences, Texas A&M University, College Station, TX USA; 4 Department of Pathobiology, School of Veterinary Medicine, University of Pennsylvania, Philadelphia, PA USA; 5 Department of Entomology, Texas A&M University, College Station, TX USA

**Keywords:** Mosquito, Dirofilariasis, Vector-borne disease, Antigen-testing, Real-time PCR

## Abstract

**Background:**

The canine heartworm *Dirofilaria immitis,* a filarioid nematode of dogs and other carnivores, is widespread in the USA and the world. Over 20 different mosquito species serve as intermediate hosts of* D. immitis*, but their contribution to transmission varies according to factors like host feeding patterns, geographic locations and climatic conditions. The yellow fever mosquito, *Aedes aegypti,* is a competent vector of *D. immitis* but is often dismissed as a vector of veterinary relevance given its anthropophilic feeding behavior. We evaluated the prevalence of *D*. *immitis* in pet dogs along the USA-Mexico border and assessed whether *Ae*. *aegypti* in the area are naturally infected with heartworm and are potentially acting as a vector.

**Methods:**

A total of 200 whole blood samples collected from pet dogs in the Lower Rio Grande Valley in south Texas from 2016 to 2019 were included in this study. Canine serum samples for *D. immitis* were tested using the DiroCHEK® Canine Heartworm Antigen Test Kit pre- and post-immune complex dissociations (ICD) and blood samples were tested using high-resolution melt (HRM) quantitative PCR (qPCR) and a probe-based qPCR. Additionally, mosquito specimens were collected and identified, and *Ae. aegypti* heads, abdomens and pools were tested using conventional PCR (cPCR) and HRM qPCR.

**Results:**

Overall, heartworm prevalence in dogs aged > 6 months was 40.8% (64/157) when the results from all testing modalities were considered. Heartworm antigen was detected in 33.5% and 40.7% of the dogs using DiroCHEK® pre- and post-ICD, respectively. By molecular screening, 20.1% of dogs tested positive with probe-based qPCR, while only one tested positive with HRM qPCR. Of the *Ae. aegypti* abdomens from blood-fed* Ae. aeygpti* tested, 20 (21.7%) from mosquitoes that fed on dogs and four (7%) from those that fed on humans tested positive for heartworm. Among *Ae. aegypti* heads from blood-fed* Ae. aeygpti*, two (1.1%) were positive based on cPCR and four (2.5%) were positive based on HRM qPCR. No *D. immitis* DNA was detected in the 208 pools of whole bodies (358 individuals) of *Ae. aegypti* gravid females.

**Conclusions:**

Our study highlights a high prevalence of heartworm in dogs in south Texas and provides evidence that *Ae. aegypti* could be contributing to heartworm transmission in canine populations in this region.

**Graphical Abstract:**

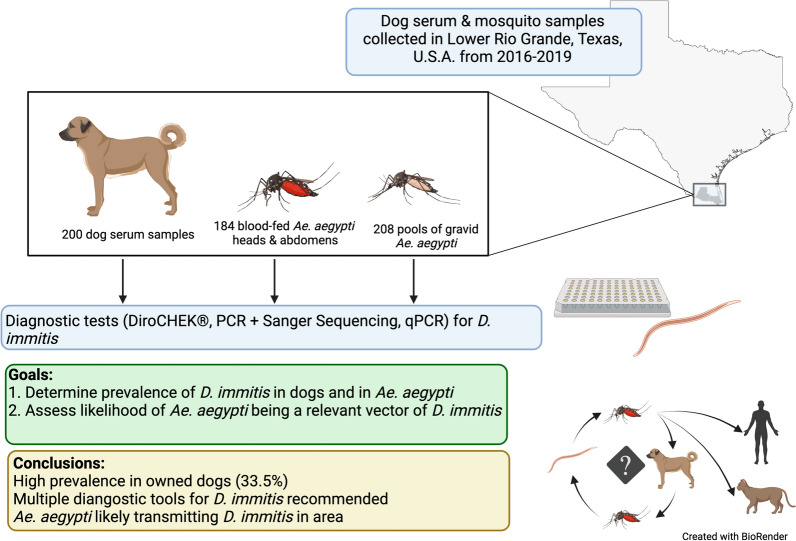

## Background

*Aedes aegypti* is an important arbovirus vector relevant to public health on the global scale, but it has not been implicated to date as being important to veterinary health due to its anthropophilic nature [1]. However, with high levels of non-human feeding reported in south Texas [2], there is the potential for *Ae. aegypti* to transmit animal pathogens, such as *Dirofilaria immitis,* the agent of dog heartworm, or allow for zoonotic bridge transmission of agents from animals to humans.

There are 23 known mosquito species or species complexes that are competent vectors of *D. immitis* in the USA [3]. The main mosquito species implicated in *D. immitis* transmission are: (i) competent as biological vectors based on laboratory infection studies; (ii) mammalophilic and utilize dogs or other canines as hosts; and (iii) distributed in areas of high heartworm prevalence [3]. Both field and laboratory evidence incriminate *Aedes vexans, A. trivittatus, A. punctipennis* and *Culex quinquefasciatus* as important vectors [3, 4]. Some mosquito species are highly competent in laboratory settings but are considered less important in nature due to infrequent feeding on dogs. *Aedes aegypti* falls into this category, as it is used as a competent model of *D. immitis* transmission in the laboratory [5–7] but is considered to be a highly anthropophilic species in most contexts [1]. Studies in the state of Florida (USA) and in Argentina have documented field-collected *Ae. aegypti* infected with *D. immitis* [8, 9], with two of 22 pools testing positive in Argentina and 17% prevalence in Florida. However, wild populations of this mosquito are rarely subjected to molecular screening for the presence of *D. immitis* DNA. Our recent study documented *Ae. aegypti* feeding more frequently on dogs than humans in south Texas [2], demonstrating the potential for high contact with canine reservoirs of *D. immitis* in some contexts. In this area, *Ae. aegypti* meets the three criteria outlined above, implicating that it is potentially an important vector of *D. immitis* in south Texas, but no studies have been conducted on mosquito vectors of heartworm in the region.

*Dirofilaria immitis* is a filarioid nematode that needs both a mosquito vector and a vertebrate host to complete its life-cycle. Microfilariae enter the mosquito during feeding on an infected dog and reach the head as third-stage larvae (L3) where they can be transmitted to new individuals [3]. Upon being bitten by an infected mosquito, the L3, the infective stage of *D. immitis,* migrate and may develop into the adult stage in dogs and cats, which often develop clinical disease. Disease severity varies, with severe disease being characterized by exercise intolerance, coughing, hemoptysis, tachypnea, dyspnea and syncope, heart failure or death [10]. Humans can also be infected with *D. immitis* via a mosquito bite, but infection is usually asymptomatic [10]. Canine heartworm disease, caused by *D. immitis,* is one of the most ubiquitous parasitic diseases of dogs in the USA with more than 100,000 cases each year [11, 12]. The cost of treatment for dogs in the USA is estimated to be US$75 million annually [12]. Disease in companion animals can be prevented through chemoprophylaxis and is recommended for dogs, cats and ferrets [13]. In the USA, canine heartworm disease is most prevalent in the southern states where mosquito vectors are present year-round [14,15].

From 2013 to 2016, canine heartworm incidence increased 17.9% in the southeastern USA, which is more than in other regions of the USA [15]. Several studies have investigated wild canids and shelter dogs and *D. immitis* in Texas [16–18], although no studies, to our knowledge, have presented results for owned dogs in the Lower Rio Grande Valley (LRGV). Many dogs in low-income communities in the LRGV live a stray lifestyle, making them unique when compared to other owned dogs in the state, whose canine heartworm prevalence was reported at 5.5% in 2008 [19] and incidence as 3.19% in 2016 [15]. The objectives of this study were to evaluate the prevalence of canine heartworm in pet dogs in low- and middle-income communities along the USA-Mexico border and to assess whether *Ae. aegypti* in the area is infected with heartworms, including disseminated infections that may support their role as a biological vector.

## Methods

### Study area

**Fig. 1:**
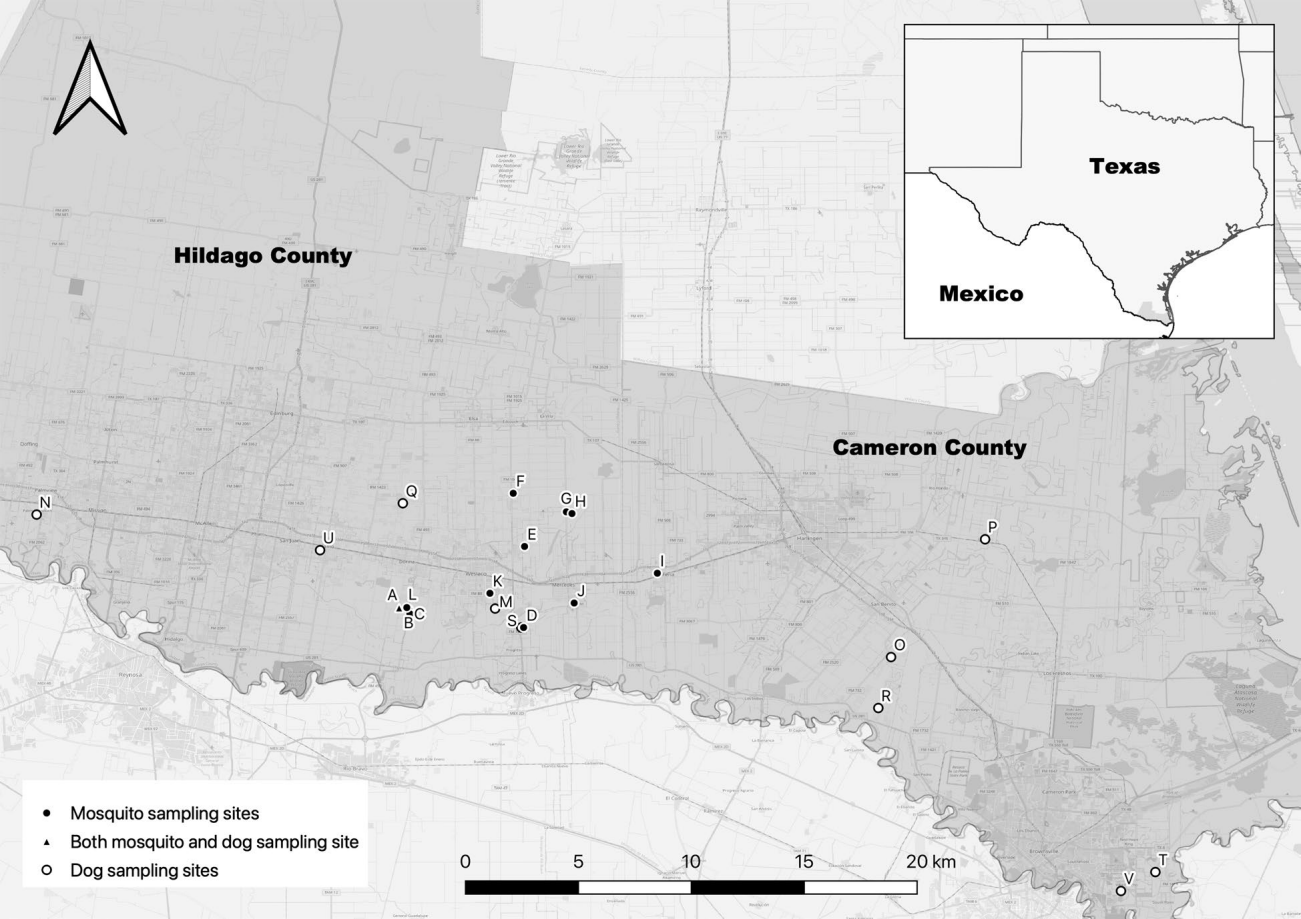
Map of sampling sites in Hidalgo and Cameron counties, Texas, USA

Samples of dog blood and mosquitoes were collected in the LRGV in south Texas along the USA-Mexico border in low- and middle-income communities, labeled communities A-V, located in Hidalgo and Cameron counties (Fig. 1). Of these communities, four (I, J, V, K) were classified as middle-income (annual household income: US$30,000–40,000) and the remainder were classified as low-income (annual household income: US$15,000–29,999) [20]. Most of these study sites are unincorporated neighborhoods called *colonias*, which are predominantly Hispanic, low-income communities where one or more city infrastructure or service is missing (e.g. poor water sanitation, lack of drainage, lack of trash collection) [21]. This lack of infrastructure can lead to a more suitable habitat for *Ae. aegypti* larvae. A detailed description of these neighborhoods has been reported previously [2, 20]. This area has a subtropical climate with a cold and dry season from November to February and a rainy season from April to October that peaks in September [22]. The annual temperature and rainfall in the LRGV ranges from 17.4 °C to 28.7 °C and 609 mm of rain, respectively, and past mosquito surveillance in these study sites documents year-round occurrence of adult female *Ae. aegypti* [20].

### Dog sampling and diagnostics

Samples of dog blood were collected via jugular or cephalic venipuncture from 340 pet dogs in eight *colonias* in the LRGV from 2016 to 2019 (Fig. 1) as previously described [23]. Of these 340 samples, 200 with adequate serum volume were selected for further testing after processing for a study focused on canine Chagas disease [23]. Of these 200 samples, extracted DNA or serum was depleted in 51 samples before all molecular testing was completed, leaving only 149 dogs that were tested using all molecular methods. Dog owners gave informed consent to sampling, and samples of 3–5 ml (from dogs > 2.3 kg) or 0.5–2 ml (from dogs < 2.3 kg) of blood were collected and divided into an EDTA tube and a serum collection tube. Age (< 6 months, 6–12 months, 1–5 years, > 5 years), breed group (herding, hound, nonsporting, other, sporting, terrier, toy, working) and sex of the dogs were recorded. All samples came from dogs aged at least 6 weeks. Rabies vaccination (RABVAC 1; Boehringer Ingelheim Vetmedica, Boehringer Ingelheim, Ingelheim, Germany) was administered to all dogs aged ≥ 3 months as incentive for participation and as a public health protective measure. Canine sampling and vaccination protocols were approved by the Institutional Animal Care and Use Committee at Texas A&M University (AUP# 20180460 and #2015-0289 CA). Blood was separated into serum, plasma and buffy coat by centrifuging at 5488 relative centrifugal field (RCF) for 8 min and DNA was extracted from the buffy coat using the E.Z.N.A. kit (Omega Bio-Tek, Norcross, GA, USA).

Serum samples were tested using the DiroCHEK® Heartworm Antigen Test Kit (Synbiotics Corporation, Zoetis, Kalamazoo, MI, USA) pre- and post-heat treatment, according to the manufacturer’s instructions. For immune complex dissociations (ICD) by heat treatment, 500 µl of serum was heated to 103 °C for 10 min in a dry heat block [24, 25], followed by centrifugation; the resulting supernatant was used as a template in the antigen test. Positive or no antigen detected (NAD) results for each sample were assessed by visual color change according to the manufacturer’s recommendation. In addition to a visual color change, optical density readings from a spectrophotometer (Epoch; BioTek Instruments Inc., Winooski, VT, USA) at 490 nm were obtained, as previously described [26].

A high-resolution melt (HRM) qPCR was used to test the extracted buffy coat DNA for *D. immitis* DNA. Previously identified primers for a 115-bp section of the mitochondrial 12S ribosomal RNA gene found in filarial worms capable of detecting multiple species [27] were used, in conjunction with a previous HRM qPCR protocol [28]. Each conventional PCR (cPCR) and HRM quantitative PCR (qPCR) plate contained ≥ 4 negative controls consisting of nuclease-free H_2_O and ≥ 1 positive controls of *D. immitis* DNA provided by the Texas A&M Parasitology Diagnostic Laboratory. We also used a simplex probe-based qPCR that targeted a 166-bp fragment of the cytochrome oxidase* c* subunit (*cox1*) gene of *D. immitis* based on a previously validated protocol [29] with the following modifications [16]: (i) oligonucleotides (Fil.COI.749-F, Fil.COI.914-R) and the FAM reporter probe (D.immi.COI.777-P) were synthesized by Invitrogen (Thermo Fisher Scientific, Waltham, MA, USA; (ii) qPCR reactions had a final volume of 20 µl containing 3.5 µl nuclease-free water, 0.5 µl of 20 µM probe, 0.5 µl of 50 µM forward and reverse primers, 10 µl of TaqMan® Multiplex Master Mix (Applied Biosystems, Thermo Fisher Scientific) and 5 µl of DNA template. The qPCR was performed on a QuantStudio 3 Real-Time PCR system (Applied Biosystems, Thermo Fisher Scientific ) using TaqMan® universal-cycling conditions, as follows: a hold stage at 95 °C for 20 s, and 40 cycles of a two-step PCR stage at 95 °C for 1 s followed by 60 °C for 20 s. All qPCR runs included a positive and negative control. The positive control consisted of DNA extracted from an adult *D. immitis* provided by the Texas A&M Parasitology Diagnostic Laboratory. The non-template negative control consisted of nuclease-free water.

### Mosquito sampling and diagnostics

Mosquito sample collection took place each month between September 2016 and December 2018 using baited BG-Sentinel 2 traps (BGS2; Biogents AG, Regensburg, Germany) and CDC Autocidal Gravid Ovitraps (AGO) (SpringStar, Inc., Woodinville, WA, USA and CDC Dengue Branch, Puerto Rico) [2, 20]. Briefly, BGS2 were baited with BG Lures outside of homes once per week for 24 h. AGOs were placed both inside and outside of homes and were checked once per week. Not all trap types were placed in all communities. Trap placement was determined by the willingness of residents to participate in the study. Mosquitoes were then identified to the species level using a dichotomous key [30] and were separated by sex. Female blood-fed mosquitoes were placed individually in nuclease-free 1.5-ml micro-centrifuge tubes and unfed females, males and gravid females each were pooled in separate tubes. Samples were stored at − 20 °C or − 80 °C until further processing.

Species identifications based on morphology made under a dissecting microscope were confirmed with a molecular protocol based on a PCR using primers for the *cox1* gene followed by Sanger sequencing [31]. Methods for DNA extraction and bloodmeal host identification have been previously described [2]. All female *Ae. aegypti* mosquitoes (*n* = 184) with identified bloodmeal hosts and with sufficient DNA were used. Briefly, mosquitoes were washed with 10% bleach to remove exogenous DNA, abdomens or heads were homogenized in 200 μl of lysis solution and DNA was extracted using the Kingfisher^™^ Flex Purification System and the MagMax Core Nucleic Acid Purification Kit (Thermo Fisher Scientific) per the manufacturer’s instructions.

We used two molecular screening methods to detect *D. immitis* DNA in mosquito samples: cPCR with Sanger sequencing and HRM qPCR. cPCR with Sanger sequencing was used to identify the presence filarial DNA as belonging to *D. immitis*, and HRM qPCR was used to detect small amounts of filarial DNA present in samples, with a positive read from one modality indicating a positive sample. Mosquito abdomens and heads were tested separately to distinguish *D. immitis* in the DNA of the bloodmeal (blood-engorged abdomen) versus disseminated L3 in the mouthpart tissues [3]. We used primers designed by Rishniw et al. [32] for the *cox1* gene that are specific to *D. immitis* for both types of PCR*.* cPCR followed by Sanger sequencing was used on DNA extracted from blood-engorged abdomens and heads. Heads only and pools of whole-bodied gravid females were tested with HRM qPCR given that the species identification results had already been confirmed using Sanger sequencing when testing the abdomens.

### Statistical analyses

Overall prevalence of *D. immitis* in dogs was calculated by categorizing as positive any dog that either tested positive by serum antigen and/or tested PCR-positive. Two prevalence values are reported, one for dogs of all ages and one for dogs of aged > 6 months. This distinction was made because the life-cycle of the parasite makes it difficult to detect infections in young dogs [25, 33] and because most epidemiology studies report prevalence only in dogs aged > 6 months. Dogs that tested positive by at least one modality were considered to be positive for analysis purposes. Unless otherwise mentioned, all values reported included dogs of all ages. We used a Z-test for two independent proportions to compare differences in prevalence between female and male dogs and between mosquitoes that fed on dogs to mosquitoes that fed on other vertebrates. For comparison of prevalence among breed group and age groups, we used the Chi-square goodness of fit tests for more than two categories.

## Results

### Heartworm prevalence in dogs

A total of 200 dog serum samples were tested using the DiroCHEK® Heartworm Antigen Test Kit. A high prevalence of *D. immitis* infection was found in sampled dogs of all ages: 33.5% (67/200) in pre-heat-treated samples and 40.7% (81/199) in post-heat-treated samples (Table 1). Only one sample tested positive pre-ICD and NAD post-ICD whereas 14 samples tested negative pre-ICD and positive post-ICD. Prevalence for heartworm in different communities ranged from 7.1% (1/14; community T) to 80% (4/5; community M). Using the HRM qPCR to assess 158 of the 200 dog blood samples (i.e. those samples that still had a sufficient quantity of DNA), we found only one (0.06%) dog positive for *D. immitis* DNA. Using the probe-based qPCR to assess the same 149 dog samples for *D. immitis* DNA, we detected 30 (20.1%) dogs that tested positive. Of these 30 dog samples with evidence of *D. immitis* DNA in the blood (buffy coat), 27 also tested positive for heartworm infection by DiroCHEK® post-ICD treatment. Combining all diagnostic modalities, 39.3% (70/178) of dogs of all ages were positive for heartworm, and 40.8% (64/157) of dogs aged > 6 months were positive.

**Table 1:** Comparative detection of *Dirofilaria immitis* in dogs from 11 communities in south Texas, USA, using different diagnostic assays

Colonia^a^	No. of samples tested	No. of samples antigen positive pre-ICD (%)	No. of samples tested	No. of samples antigen positive post-ICD (%)	No. of samples tested	No. of samples HRM qPCR positive (%)	No. of samples tested	No. of samples probe-based qPCR positive
A	17	6 (35.3)	17	8 (47.1)	17	0 (0.0)	17	4 (23.5)
M	5	1 (20.0)	5	4 (80.0)	5	0 (0.0)	5	1 (20.0)
N	7	3 (42.9)	7	3 (42.9)	7	0 (0.0)	7	2 (28.6)
O	25	10 (40.0)	25	11 (44.0)	25	0 (0.0)	24^b^	7 (29.2)
P	17	10 (58.8)	17	10 (58.8)	12^b^	0 (0.0)	12^b^	3 (25.0)
Q	42	9 (21.4)	42	10 (23.8)	34^b^	1 (2.9)	30^b^	5 (16.7)
R	19	9 (47.4)	19	10 (52.6)	19	0 (0.0)	13^b^	4 (30.8)
S	23	12 (52.2)	23	15 (65.2)	0^b^	NA	0^b^	NA
U	15	0 (0.0)	14^a^	2 (14.3)	15	0 (0.0)	15	0 (0.0)
V	16	6 (37.5)	16	7 (43.8)	16	0 (0.0)	16	3 (18.8)
T	14	1 (7.1)	14	1 (7.1)	8^b^	0 (0.0)	10^b^	1 (10.0)
Total	200	67 (33.5)	199	81 (40.7)	158	1 (0.06)	149	30 (20.1)

*cPCR* Conventional PCR,* HRM* high-resolution melt,* ICD* immune complex dissociation,* NA* not available,* qPCR* quantitative PCR

^a^Unincorporated neighborhoods that are predominantly Hispanic, low-income communities

^b^Sample sizes are reduced in later testing methods due to insufficient sample remaining

In total, 178 dogs were tested using all modalities, of which 96 were male and 82 were female. There was no significant difference in prevalence of *D. immitis* between sexes, with 67.7% (64/96) of males testing positive and 52.4% (43/82) of females testing positive (*χ*^2^ = 3.71,* df* = 1, *P* = 0.054) when dogs of all ages were considered. The number of positive dogs varied by age group (*χ*^2^ = 8.34,* df* = 3, *P* = 0.040), with dogs aged 6–12 months showing the highest level of prevalence followed by dogs aged between 1 and 5 years. However, the number of positive dogs did not significantly vary by breed group (*χ*^2^ = 9.94* df* = 7, *P* = 0.192) (Table 2). All analyses, with the exception of overall prevalence in dogs aged ≥ 6 months, included dogs of all ages (i.e. aged ≥ 6 weeks).

**Table 2:** Comparison of dog breed and age groups in dogs of all ages that tested positive for *D. immitis* by at least one testing modality

Category	No. of dogs tested	No. of dogs positive for *D. immitis* (%)
*Breed group*		
Herding	24	12 (50.0)
Hound	3	2 (66.6)
Nonsporting	3	1 (33.3)
Other	6	0 (0.0)
Sporting	22	10 (45.5)
Terrier	38	10 (26.3)
Toy	65	29 (44.6)
Working	17	6 (35.3)
Total	**178**	**70 (39.3)**
*Age group*		
< 6 months	21	6 (28.5)
6–12 months	18	10 (55.6)
1–5 years	116	50 (43.1)
> 5 years	23	4 (17.4)
Total	**178**	**70 (39.3)**

Only dogs that were tested by all testing modalities were included in the analysis

### *D. immitis *DNA in *Ae. aegypti*

We used cPCR with Sanger sequencing to test abdomens of blood-fed* Ae. aeygpti* (*n* = 184) with the blood host identified for *D. immitis* presence; 25 (13.6%) of these abdomens from mosquitoes with identified vertebrate bloodmeal tested positive for *D. immitis* DNA. *Dirofilaria immitis* was more commonly found (*χ*^2^ = 9.07,* df* = 1, *P* = 0.003) in the abdomens of mosquitoes that had fed on dogs (21.7%; 20/92) than in those that fed on cats, sheep, human and other vertebrates (5.4%; 5/92) (Table 3). The heads of four mosquitoes tested positive for *D. immitis* DNA, including three heads from mosquitoes that fed on dogs and one head from a mosquito that fed on a cat. No *D. immitis* DNA was found in the 208 pools of gravid females (358 individuals). The obtained 25 sequences had 97–100% similarity to the reference *D. immitis* sequence (GenBank Accession numbers: OP681144, OP681145 ).

**Table 3:** Results of conventional and high-resolution melt PCR analysis for *D. immitis* DNA in the abdomens and heads of *Aedes aegypti* collected in south Texas, USA

Host	cPCR^a^			HRM qPCR^a^	
	No. of samples tested	Abdomen (%)	Head (%)	No. of samples tested	Head (%)
Cat	22	0 (0)	1 (4.5)	17	1 (5.9)
Dog	92	20 (21.7)	1 (1.1)	83	3 (3.6)
Sheep	3	1 (33.3)	0 (0)	1	0 (0)
Human	57	4 (7.0)	0 (0)	49	0 (0)
Other vertebrates^b^	10	0 (0)	0 (0)	7	0 (0)
Total	184	25 (13.6)	2 (1.1)	157	4 (2.5)

^a^Number of abdomens and heads with bloodmeal testing positive for *D. immitis* DNA, with the percentage positivity in parentheses

^b^Includes chickens, birds, opossums, lizards and pigs

## Discussion

Our findings suggest that there is a high prevalence of canine heartworm infection in dogs from south Texas along the USA-Mexico border, with an infection prevalence of 40.8% in dogs from diverse communities, most of which consist of low-income *colonias*. Texas has been indicated to be an area of high canine heartworm incidence [34]. In pet dogs that have regular access to veterinary care, 23,489 positive cases were reported in 2016, 28,320 cases in 2017, 31,252 cases in 2018 and 35,656 cases in 2019 [13]. These data confirm a previously established trend of increasing prevalence of canine heartworm infection in Texas, with an increase of 17.23% from 2013 to 2016 [15]. Shelter dogs in the same area (i.e. Edinburg, TX) were documented in a previous study to have a lower prevalence of *D. immitis* than our samples, with 19/33 (20.9%) dogs being positive [35]; however, the diagnostic test used was a commercial, point-of-care enzyme-linked immunosorbent assay (ELISA) kit and did not include ICD by heat treatment, which may explain the discordance. In general, shelter dogs tend to have higher canine heartworm prevalence than owned dogs [36, 37]. However, shelter dogs having a lower incidence of infection than owned dogs from middle- and low-income communities may be explained by the lower socioeconomic status (SES) of sampled *colonias*, as low SES has been linked with higher prevalence of *D. immitis* in the state of North Carolina (USA) [38], or by other variables, such as sampling year or location. Moreover, owned dogs in the sampled area of the present study generally are kept outside and have a stray lifestyle. Therefore, it is likely that many of these owned dogs were not receiving veterinary care or macrocyclic-lactone heartworm preventives as two-thirds of dogs in the USA are not receiving preventative care [15]. Even for dogs receiving preventive care, failures of heartworm preventative measures have been reported in endemic states, such as Texas [39]. These same communities also have many stray dogs which likely have even higher heartworm prevalence rates than the population of dogs sampled in this study.

The American Heartworm Society recommends both antigen testing and microfilariae testing of all canine samples [33], but microfilariae tests are not routine procedure. Consistent with our own previous results [16, 42], antigen and microfilaria testing by qPCR in the present study gave different estimates of prevalence. Antigen tests work well at diagnosing infection with higher numbers of female *D. immitis* worms but are less sensitive for male-only infection with ≤ 3 females [40]. Moreover, antigen tests are more effective at identifying female worms that are > 6 months of age, making infection difficult to diagnosis in dogs aged < 6 months as it takes 50–58 days post-infection for L3 to develop into adults [41]. qPCR is a reliable and specific diagnostic tool for *D. immitis* [42] and the use of multiple diagnostic tests has been shown to improve diagnostic performance [29, 43, 44]. Two earlier studies found that qPCR detected a higher prevalence rate than antigen testing alone [29, 45], similar to our results as three dogs in the present study were considered to be *D. immitis* microfilaria positive and NAD before and after ICD. However, to our knowledge, the sensitivity of probe-based qPCR and HRM qPCR for the detection of nematodes have not been compared, making it difficult to comment on the differences between the two types of qPCR used in the present study. The increased detection of antigen post-ICD was expected given the study population, which comprised dogs from an endemic area, many of which had no history of heartworm prevention. Also, antigen tests are recommended for dogs aged > 6–7 months [25, 33]. In the present study, six dogs aged < 6 months tested positive in the post-ICD treatment, which is not surprising since heat treatment allows for earlier detection of infection [46]. Accordingly, we present our results both including and excluding dogs aged < 6 months. We saw a trend of highest infection prevalence in dogs aged 6–12 months, with a decrease of prevalence of infection after 1 year of age. This decrease could be explained by the potential fatality of dogs with untreated infection, with older dogs likely to have been infected longer and therefore have more advanced disease.

We found that of the *Ae. aegypti* abdomens with bloodmeal identified from *Ae. aegypti* that fed on dogs, 21.7% contained *D. immitis* DNA, suggesting that *Ae. aegypti* in these communities are frequently exposed to microfilaremic dogs. Some mosquitoes that fed on other vertebrates also tested positive for *D. immitis,* which may be explained by infections in other hosts (e.g. cats) and/or by a previous feeding on dogs that was not the most recent bloodmeal (i.e. mixed bloodmeal from > 1 vertebrate species)*.* We also found four *Ae. aegypti* heads positive for *D. immitis* DNA out of the 157 tested, a rare observation which has only been documented for wild populations in the state of Florida (USA) [8]. L3, the infective stage of *D. immitis*, move to the mosquito head and enter the salivary gland; saliva, hemolymph and L3 move to the skin then enter the host body through the mosquito bite wound [47]. Therefore, the detection of *D. immitis* DNA in mosquito heads represents the presence of L3, providing evidence of the suitability of the *Ae. aegypti* as a biological vector. The prior findings of *Ae. aegypti* being a competent vector of *D. immitis* based on laboratory studies [6, 7] combined with the results presented in this study suggest that *Ae. aegypti* likely contributes to canine heartworm transmission in south Texas. Indeed, potential transmission has been assumed of a mosquito species if a positive result was found in a competent species [38]. In their study, Palmer et al. noted that large worm burdens in *Ae. aegypti* can lead to destruction of the excretory system and therefore mortality, a phenomenon that may be occurring in *Ae. aegypti* in the LRGV as no gravid females tested positive for *D. immitis* DNA [48]. As the relative role of different mosquito species in the transmission of *D. immitis* to dogs is rarely measured, little is known on the role of *Ae. aegypti* contribution to transmission. In an earlier study, Paras et al. concluded that a mosquito can be considered to be a relevant vector based on its abundance and infection prevalence, reporting *Ae. albopictus* as an important vector based on a prevalence of approximately 15% calculated based on pool testing [49]. Still, a lower prevalence (e.g. 0.30% in *Aedes albopictus* [38], 0.7% in *Culix erraticus* [50], 2.3% in *Ae. vexans* [51]) has been reported for accepted heartworm vectors. Given that we found a prevalence of 13.6% in abdomens and 2.5% in heads, it reasonable to conclude that *Ae. aegypti* has a sufficiently high prevalence to be a vector of *D. immitis.*

While our previous study documented that these populations of *Ae. aegypti* fed 50% of the time on dogs, we also documented 31% feeding on humans and 12% feeding on cats [2]. This result suggests the potential for *Ae. aegypti* to also contribute as a bridge vector species, allowing spill-over transmission of *D. immitis* from dogs to humans. Human infections with *D. immitis* have been reported in North and South America, Europe, Asia and Australia, although most cases are asymptomatic [10]. When symptoms do occur, they include cough, chest pain, fever, hemoptysis and pleural effusion [10]. Infection is difficult to diagnose and may be underreported [52]. In the USA, fewer than 120 cases have been reported since 1941 [2], with the highest reported incidence in humans occurring in the southeastern region, with 23 reported cases as of 2005 [52]. At least one case of *D. immitis* infection in a human patient has been reported in Texas [53]. Infection in dogs is highest in the southeast USA [19] and is increasing in prevalence in this region [15]. It should be noted that this region overlaps with the establishment of *Ae. aegypti* in many regions of the southern USA [11, 19, 54]

## Conclusions

In the present study, the prevalence of *D. immitis* infection in owned dogs in low- and middle-income communities in the LRGV, Texas, was high (40.8%). As prevalence estimates varied based on the diagnostic modality used, our results further support the use of integrated immunodiagnostic and molecular tests to determine heartworm prevalence. The finding of *D. immitis* DNA in the heads of *Ae. aegypti* provides evidence that this mosquito could be contributing to heartworm transmission in canine populations and potential spill-over to human populations. However, further study is needed to inform the relative role of *Ae. aegypti* and other mosquito species in transmitting *D. immitis* in the region.

## Data Availability

Data is available upon request.

## Abbreviations

cPCR: Conventional PCR
HRM: High-resolution melt
ICD: Immune complex dissociation
LRGV: Lower Rio Grande Valley
NAD: No antigen detected
qPCR: Quantitative PCR
